# Lipoxin A4 and Platelet Activating Factor Are Involved in *E. coli* or LPS-Induced Lung Inflammation in CFTR-Deficient Mice

**DOI:** 10.1371/journal.pone.0093003

**Published:** 2014-03-26

**Authors:** Haiya Wu, Jun Yang, Emily M. Su, Ling Li, Caiqi Zhao, Xi Yang, Zhaowei Gao, Mengyao Pan, Peiyu Sun, Wei Sun, Yiyi Jiang, Xiao Su

**Affiliations:** 1 Key Laboratory of Molecular Virology & Immunology, Unit of Respiratory Infection and Immunity, Institut Pasteur of Shanghai, Chinese Academy of Sciences, Shanghai, China; 2 Department of Entomology, University of California Davis, Davis, California, United States of America; 3 Cardiovascular Research Institute, University of California San Francisco, San Francisco, California, United States of America; University of Torino, Italy

## Abstract

CFTR (cystic fibrosis transmembrane conductance regulator) is expressed by both neutrophils and platelets. Lack of functional CFTR could lead to severe lung infection and inflammation. Here, we found that mutation of CFTR (*F508del*) or inhibition of CFTR in mice led to more severe thrombocytopenia, alveolar neutrocytosis and bacteriosis, and lower lipoxin A4/MIP-2 (macrophage inhibitory protein-2) or lipoxin A4/neutrophil ratios in the BAL (bronchoalveolar lavage) during acute *E. coli* pneumonia. *In vitro*, inhibition of CFTR promotes MIP-2 production in LPS-stimulated neutrophils; however, lipoxin A4 could dose-dependently suppress this effect. In LPS-induced acute lung inflammation, blockade of PSGL-1 (P-selectin glycoprotein ligand-1) or P-selectin, antagonism of PAF by WEB2086, or correction of mutated CFTR trafficking by KM11060 could significantly increase plasma lipoxin A4 levels in *F508del* relevant to wildtype mice. Concurrently, *F508del* mice had higher plasma platelet activating factor (PAF) levels and PAF-AH activity compared to wildtype under LPS challenge. Inhibiting hydrolysis of PAF by a specific PAF-AH (PAF-acetylhydrolase) inhibitor, MAFP, could worsen LPS-induced lung inflammation in *F508del* mice compared to vehicle treated *F508del* group. Particularly, depletion of platelets in *F508del* mice could significantly decrease plasma lipoxin A4 and PAF-AH activity and deteriorate LPS-induced lung inflammation compared to control *F508del* mice. Taken together, lipoxin A4 and PAF are involved in *E. coli* or LPS-induced lung inflammation in CFTR-deficient mice, suggesting that lipoxin A4 and PAF might be therapeutic targets for ameliorating CFTR-deficiency deteriorated lung inflammation.

## Introduction

Our previous studies have shown that CFTR expressed by neutrophils and platelets plays an important role in mediating LPS-induced acute lung inflammation [1]–[3]. Patients with cystic fibrosis (CF, caused by CFTR gene mutation) suffer from recurrent lung infection and inflammation manifested by higher neutrophils and other inflammatory profiles and lower lipoxin A4 levels in the lung [4]. Lipoxin A4 is an anti-inflammatory lipid mediator, which is generated during platelet-neutrophil interaction [5], [6]. Systemically, neutrophil-platelet aggregation and platelet P-selectin expression are increased in CF patients [7], [8].

So far, the role of CFTR in acute lung infection has not been very clear. In this study, we approached an *E. coli* pneumonia *F508del* mouse model to test whether mutation of CFTR would affect blood platelet counts, lung bacterial titers, and inflammatory profiles. Since the CFTR inhibitors were invented, many researches have used CFTR inhibitors to mimic the dysfunctional state of CFTR mutation or deletion [9], [10]. Therefore, two different specific CFTR inhibitors (MalH-2 and CFTR_inh_-172) were used to test whether inhibition of CFTR would worsen lung infection and inflammation, especially reduce lung lipoxin A4 levels as seen in *F508del* mice.

We have reported that CFTR inhibition augmented production of MIP-2 in neutrophils in an NF-kB-dependent manner [1]. Considering p38MAPK and PI3K are two important signaling in mediating cytokine production, we tested whether application of p38MAPK or PI3K inhibitor to neutrophils would affect CFTR inhibition-mediated MIP-2 production. Furthermore, we also tested whether administration of lipoxin A4 to neutrophils would affect CFTR inhibition-mediated MIP-2 production. These findings would demonstrate the protective effects of lipoxin A4 on inflammatory responses in the neutrophils.

During lung inflammation, dysfunction of CFTR promotes neutrophil-platelet interaction, by which synthesis of lipoxin A4 initiates [6]–[8]. Coupling PSGL-1 (in neutrophils) with P-selectin (in platelets) promotes neutrophil transmigration [2], [11]. We have reported that interruption of neutrophil-platelet interaction by blockade of PSGL-1, PAF, or correction of mutated CFTR trafficking could lessen LPS-induced acute lung inflammation [2]. Therefore, we should test whether blockade of PSGL-1, P-selectin, PAF, or correction of mutated CFTR trafficking would affect lipoxin A4 synthesis and lessen lung inflammation.

Blockade of PAF attenuates LPS-induced lung inflammation in the CFTR-deficient mice, suggesting that PAF is an important proinflammatory mediator [2]. Study has shown that platelets are able to secrete PAF-AH [12], which is a rate-limiting enzyme for hydrolysis of PAF. PAF-AH is required for suppressing the development of sepsis [13]. In this study, we hypothesized that CFTR deficiency would affect PAF levels and PAF-AH activity systemically. Inhibition of PAF-AH activity (reducing hydrolysis of PAF) would promote PAF-mediated lung inflammation induced by LPS challenge. To test this hypothesis, we measured plasma PAF levels and PAF-AH activity in LPS-challenged wildtype and *F508del* mice. We also pretreated *F508del* mice with PAF-AH inhibitor (MAFP) and then intratracheally challenged them with LPS. The aim of these experiments is to determine whether PAF is involved in the lung inflammation worsened by CFTR deficiency.

Lipoxin A4 is inducible during neutrophil-platelet interaction [6] and platelets contribute to PAF-AH activity [12], suggesting that platelets may be required for dampening lung inflammation. As such, depletion of platelets would reduce plasma lipoxin A4 levels, activity of PAF-AH, and worsens lung inflammation in CFTR-deficient mice. To elucidate this rationale, we used antibody to deplete platelets and challenged the *F508del* mice with LPS to measure plasma lipoxin A4 levels, PAF-AH activity, and extent of lung inflammation.

Collectively, the major objective of this study was to elucidate that whether lipoxin A4 and PAF are involved in *E. coli* or LPS-induced lung inflammation in the CFTR-deficient mice. The findings will provide us novel therapeutic strategies for ameliorating CFTR-deficiency deteriorated lung inflammation.

## Materials and Methods

### Reagents

CFTR_inh_-172 (Thiazolidinone CFTR inhibitor, 3-[(3-Trifluoromethyl) phenyl]-5-[(4-carboxyphenyl) methylene]-2-thioxo-4-thiazolidinone) was purchased from EMD Millipore (Billerica, MA). MalH-2 (di-sulfonate Glycine Hydrozide), a water soluble CFTR inhibitor, was provided by Verkman AS (UCSF). SB203580 and Wortmannin were purchased from Calbiochem (San Diego, CA). LPS (*E. coli* 0111:B4) was from Sigma-Aldrich (St. Louis, MO). Lipoxin A4 analog, Methyl Arachidonyl Fluorophosphonate (MAFP), and PAF acetylhydrolase assay kit were from Cayman Chemical (Ann Arbor, MI). Purified NA/LE Rat Anti-Mouse CD162, Rat (LEW) IgG1, κ; and purified NA/LE rat IgG1, κ isotype control antibodies; PE-conjugated PSGL-1 antibody or PE-conjugated isotype control antibody were purchased from BD Pharmingen (San Diego, CA). WEB2086 (a platelet activating factor receptor antagonist) was from Tocris Bioscience (Ellisville, MO). PAF C16 was purchased from Avanti Polar Lipids Inc (Alabaster, AL).

### Administration of CFTR Inhibitors

Mice were intraperitoneally injected (ip) with MalH-2 (dissolved in PBS, 3 mg/kg) or CFTR_inh_-172 (dissolved in DMSO, 3 mg/kg) 15∼20 min before intratracheal challenge with *E. coli* or LPS to establish lung inflammation mouse models.

### *E. coli* Pneumonia and LPS-induced Acute Lung Inflammation Models

Eight to ten-week old CD1 wild-type and CF mice (targeted *F508del* gene replacement, obtained from Professor A. Verkman, University of California San Francisco) were used for these studies [1], [2]. Anesthesia was induced with an ip injection of a mixture of ketamine (90 mg/kg) and xylazine (10 mg/kg). The Committee on Animal Research of the University of California, San Francisco and Institut Pasteur of Shanghai, Chinese Academy of Sciences approved all the protocols.

A previously developed direct visualization instillation (DVI) method was used to instill LPS into the airspaces of the lung [14]. The LPS dosage (5 mg/kg) was selected aiming to induce a robust lung inflammation and injury at 24 h as previously reported [1], [2] and no mice died at this dosage. For establishing *E. coli* pneumonia, 10^7^ cfu of *E. coli* were instilled into the airspaces of the lung as reported before [15], [16]. *E. coli* pneumonia and LPS-induced acute lung inflammation mouse models were followed for 4 and 24 respectively. Vital signs of each mouse were observed timely. At the end of experiment, mice were first anesthetized and then sacrificed by cervical dislocation.

### Platelet Depletion

For the platelet depletion experiments, we used a rabbit, anti-mouse serum (25 μg, intravenous injection) delivered 2 hours prior to intratracheal challenge of LPS (5 mg/kg, Sigma) [17]. Normal rabbit serum was used as a control antibody in the depletion protocol.

### Bronchoalveolar Lavage (BAL), Measurement of BAL Protein Levels and Neutrophil Counts

BAL was performed as previously described [2]. Protein concentration in the BAL was determined by a Bio-Rad protein assay (Bio-Rad Laboratories, Hercules, CA). BAL cell smear was made using cytospin (Shandon Inc, Pittsburgh, PA). The slides were visualized using Wright-Giemsa staining (Fisher Scientific Co., Middletown, VA). The number of neutrophils was determined by a certified laboratory technologist.

### *E. coli* Culture

*E. coli* serotype K1 was originally isolated from the blood of a patient with biliary sepsis [18]. The methods used to passage, store, and amplify the bacteria have been described elsewhere [18]. To monitor *E. coli* growth, serially diluted BAL or lung homogenate samples were inoculated on LB agar plate. After 24 h incubation at 37°C, the colonies of *E. coli* were counted.

### ELISA

MIP-2 and Lipoxin A4 in the plasma, BAL, or supernatant of media were measured by ELISA kits (MIP-2, R & D Systems Inc, Minneapolis, MN; lipoxin A4, Neogen Corporation, Lexington, KY) according to the manufacturers’ instructions.

### Measurement of Platelets in Blood

A sample of blood was placed in EDTA-coated vials (BD Microtainer) and Hemavet analyzer (Drew Scientific Inc, Dallas, TX) was used to generate blood platelet counts.

### Neutrophil Isolation and Culture

The procedure of neutrophil isolation has been described previously [1]. To validate the purity of isolated neutrophils, we prepared the isolated cells on slides by cytospin and stained them with Hema 3 staining. Morphology demonstrated that about 93% were neutrophils with ring-shaped nuclei (feature of immature neutrophils) and the left cells were lymphocytes. The isolated neutrophils were resuspended in RPMI 1640 medium supplemented with 2 mM glutamine, 100 U/ml penicillin, 100 μg/ml streptomycin, and 10% FCS (v/v). The final concentration of neutrophils was 10^6^/ml in 200 μl media. MalH-2 (100 μM) was added 30 min before LPS (30 μM, which induced a higher proinflammatory cytokine production) stimulation. After 12 h, the supernatant was aspirated, centrifuged, and stored at −70°C. Trypan blue exclusion was used to detect viability of neutrophils after 12 h culture.

### Plasma PAF-AH Activity Assay

PAF-AH activity in plasma was measured using PAF acetylhydrolase assay kit. Briefly, 10 μl plasma and 10 μl DTNB (5,5′-dithiobis-(2-nitrobenzoic acid) were added into 96-well plate. The reactions were initiated by adding 200 μl substrate solution (2-thio-PAF). The absorbance was read every minute at 405 nm using a plate reader.

### PAF Measurement by High Performance Liquid Chromatography-mass Spectrometry (HPLC-MS)

We added 200 μl of plasma to 400 μl acetonitrile in an eppendoff tube. The tubes were then centrifuged at 14,000 rpm at 4°C for 10 minutes. The supernatant were then transferred to the vials for the LC/MS/MS analysis. Data were collected on a 4000 QTrap tandem mass spectrometer (Applied Biosystems Instrument Corporation, Foster City, CA) equipped with an electrospray source (Turbo V). At first, the tandem mass spectrometer was operated under negative ESI mode to be able to detect not only PAF C16 but also the possible interference suggested by Owen *et al* [19]. The transitions are −508 →−59 (PAF C16), −508→−255 (PFPC, palmitoyl-formyl-glycerophosphocholine), −508→−283 (stearoyl-lyso phosphocholine) respectively. Then the liquid chromatography (LC) conditions were optimized to separate these three compounds by injecting the PAF C16 spiked plasma and PAF C16 standard. The liquid chromatography system used for analysis was an Agilent 1,200 SL liquid chromatography series (Agilent Corporation, Palo Alto, CA). The autosampler was kept at 4°C.

Liquid chromatography was performed on an Ascentis Express C18 2.1×150 mm, 2.7 μm column. Mobile phase A was water with 10% acetonitrile and 0.1% formic acid. Mobile phase B consisted of 90% acetonitrile with 0.1% formic acid. The column was eluted at a flow rate of 400 μl/min with a gradient of 60% B to 100% B over 5 min; hold at 100% B for 1 min; return to 60% B over 0.1 min; hold at 60% B for 4 min to regenerate the column.

After the separation, the mass spectrometer was performed under positive mode to obtain a more sensitive measurement. The transition used is 524.3→184. All the acquired data were quantified using Analyst 1.5.1 software (AB Sciex, Foster City, CA) according to the calibration curve generated by the standards range from 2.5 ng/mL to 2.5 ug/ml PAF C16.

### Statistical Analysis

**Figure 1:**
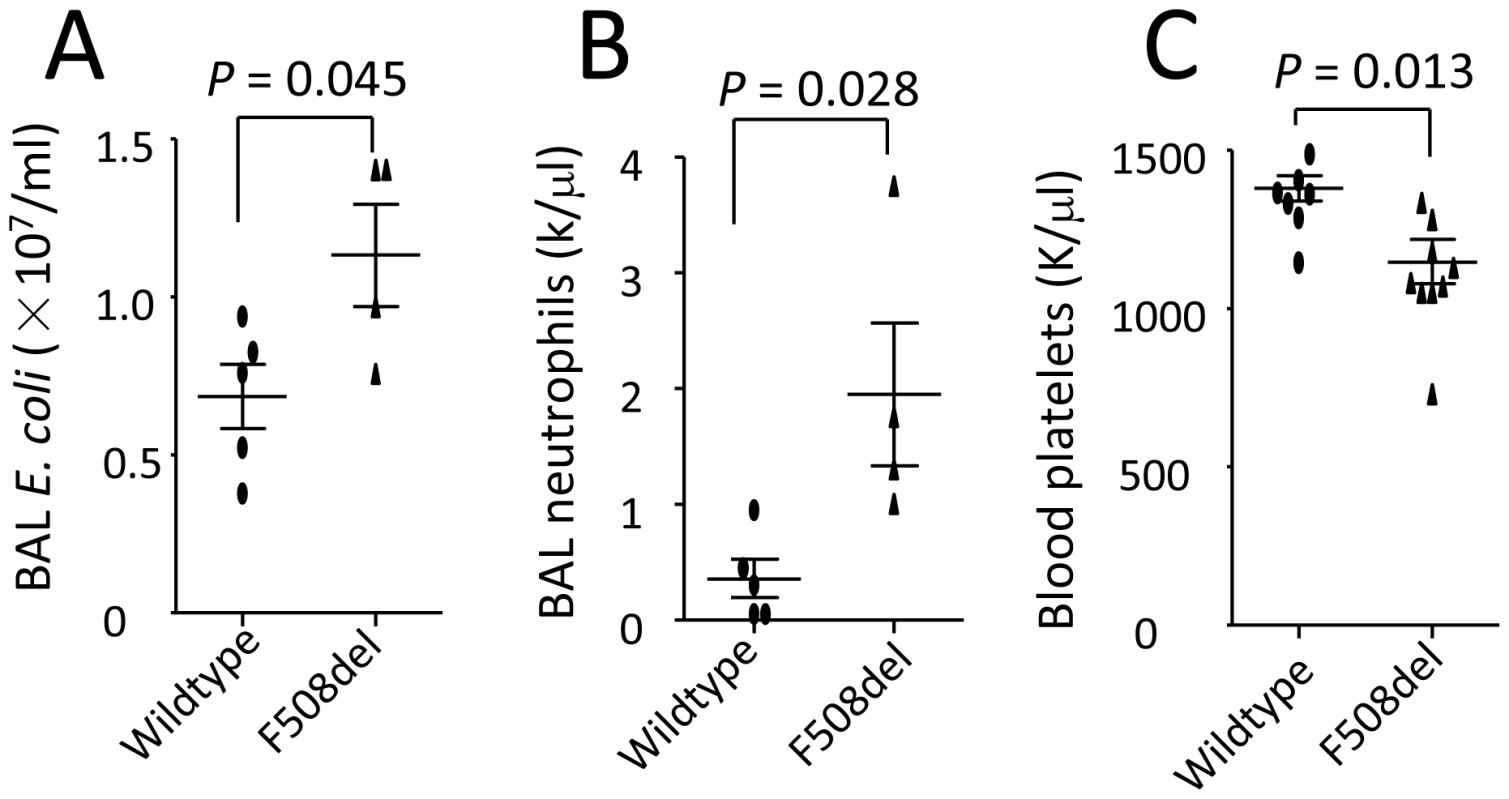
Mutation of CFTR worsens lung infection, alveolar neutrocytosis, and thrombocytopenia. Wildtype and *F508del* mice were intratracheally challenged with *E. coli* (10^7^ cfu) and killed at 4 h. BAL was performed to culture bacteria and count neutrophils. Blood was withdrawn to count platelets. **A**. BAL *E. coli* counts. **B**. BAL neutrophil numbers. **C**. Blood platelet counts. Values are presented as mean ± SD. Sample size (each dot represents one sample) and *P* values are indicated in each panel.

**Figure 2:**
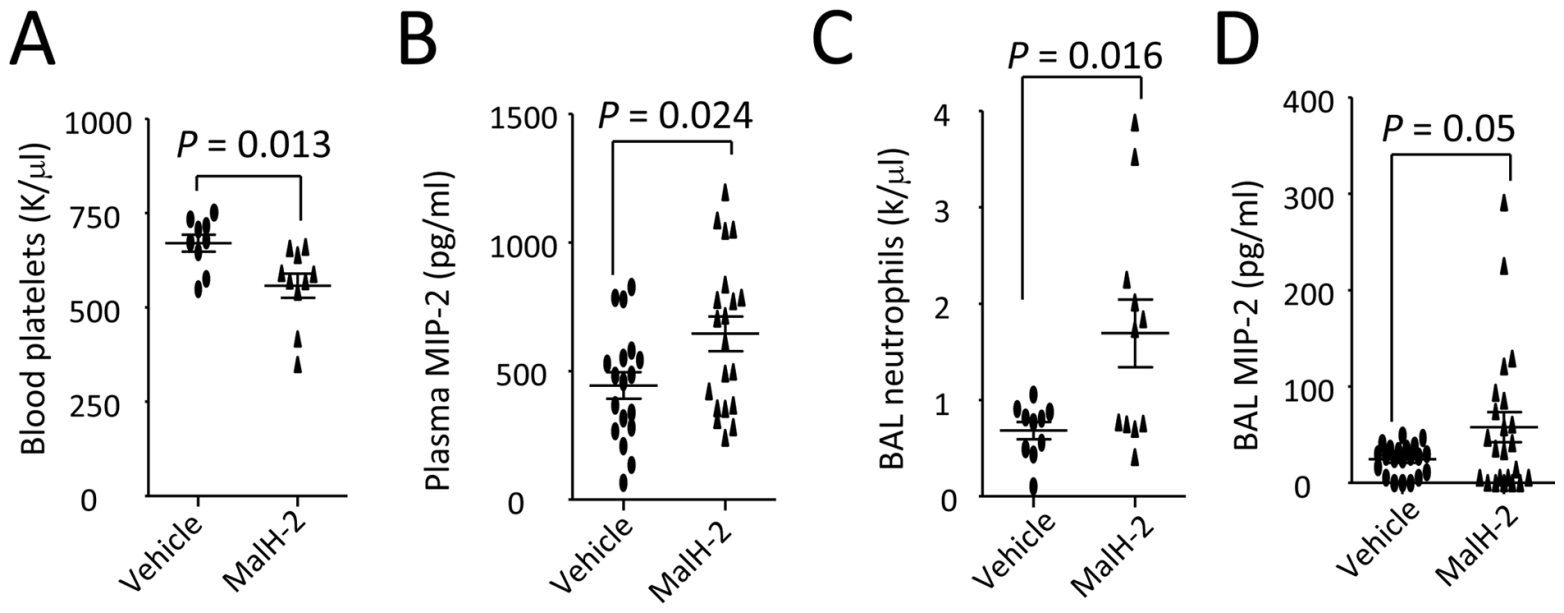
CFTR inhibition by MalH-2 affects blood platelet counts, plasma MIP-2 levels, and parameters of lung inflammation in *E. coli* pneumonia. Wildtype mice were divided into two groups to receive an ip of either PBS or MalH2 (3 mg/kg), then intratracheally challenged with *E. coli* (10^7^ cfu). At 4 h, mice were killed to collect plasma and BAL and measure the relevant parameters. **A**. Blood platelet counts. **B**. Plasma MIP-2 levels. **C**. BAL neutrophils. **D**. BAL MIP-2 levels. Values are presented as mean ± SD. Sample size and *P* values are indicated in each panel.

**Figure 3:**
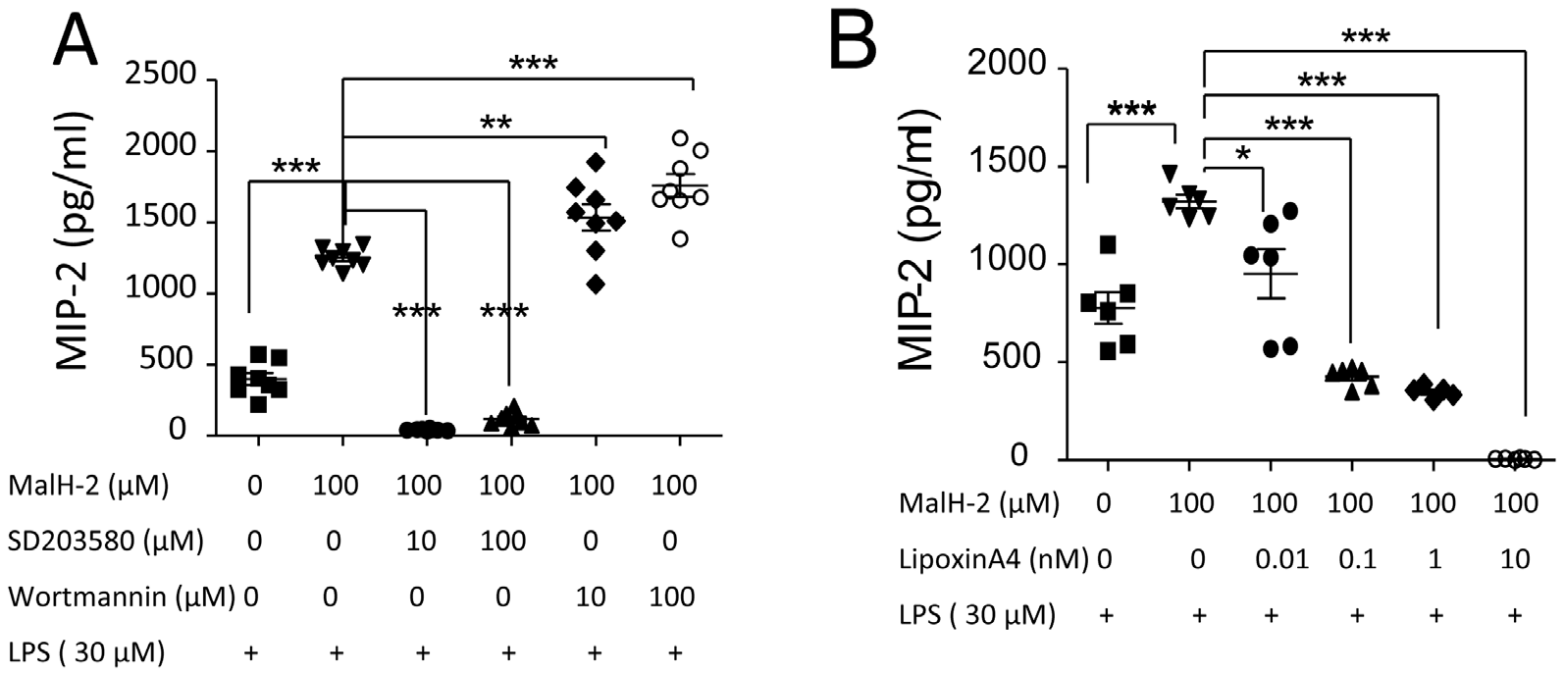
The effect of lipoxin A4 on MIP-2 production in LPS-stimulated neutrophils. **A**. Inhibition of CFTR by MalH-2 affect MIP-2 production in LPS stimulated neutrophils. The pretreatment with p38MAPK (SB203580) or PI3K (Wortmannin) inhibitor influence MIP-2 production in LPS stimulated neutrophils pretreated with MalH2. **P<0.01; ***P<0.001. **B**. The effects of lipoxin A4 on MIP-2 production in LPS-stimulated neutrophils pretreated with MalH2. *P<0.05; ***P<0.001. Values are presented as mean ± SD. Sample size and *P* values are indicated in each panel.

Statistical analysis was performed using GraphPad Prism 5.0 (GraphPad Software, San Diego, CA). Figures 1, 2 and the other work were analyzed by Student’s t test except that Figure 3 was analyzed by one-way analysis of variance (ANOVA) with *post hoc* Bonferroni test, Figure 5 by two-way ANOVA, and Figure 6B, 6C, and 7 C by repeated measures two-way ANOVA. Significance was set at *P*<0.05. Results are presented as means ± SD.

**Figure 4:**
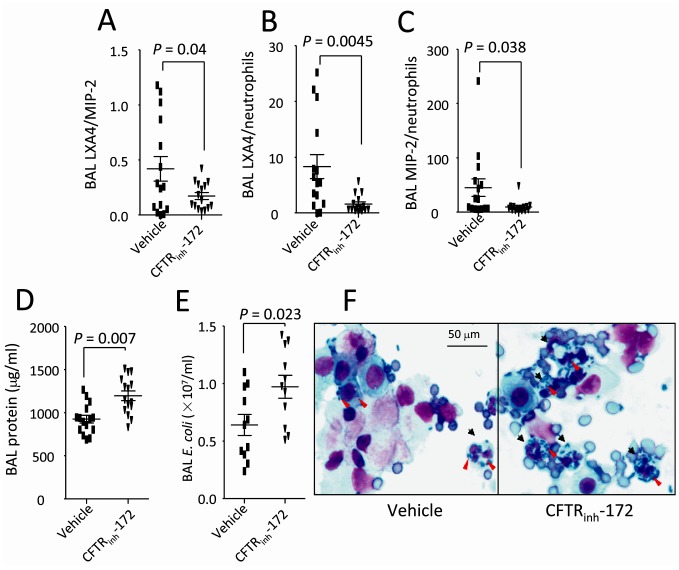
CFTR inhibition by CFTR_inh_-172 affects lipoxin A4, inflammatory parameters, and *E. coli* number in BAL. Mice were pretreated with either vehicle or CFTR_inh_-172 and then intratracheally challenged with *E. coli* (10^7^ cfu). At 4 h, mice were killed to collect BAL and measure the relevant parameters. **A**. BAL lipoxin A4/MIP2 ratio. **B**. BAL lipoxin A4/neutrophils ratio. **C**. BAL MIP-2/neutrophils ratio. **D**. BAL protein levels. **E**. BAL *E. coli* numbers. **F**. Cytological changes of BAL proinflammatory cells and *E. coli* infection. Black arrows indicate infected cells; Red arrows denote *E. coli*. Wright’s staining, magnification×100. Values are presented as mean ± SD. Sample size and *P* values are indicated in each panel.

**Figure 5:**
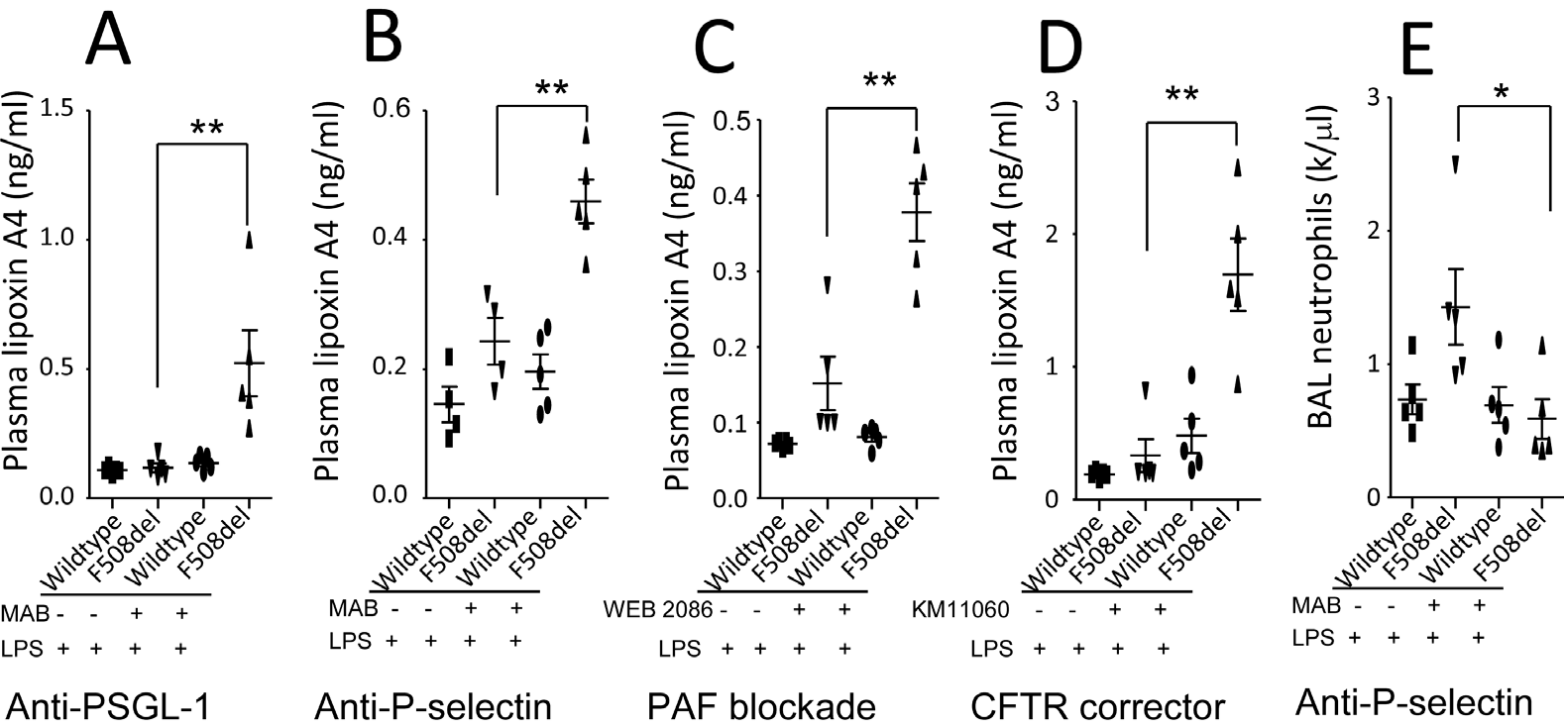
Effects of blockade of PSGL-1, P-selectin, PAF, or correction of mutated CFTR trafficking on plasma lipoxin A4 in LPS-induced lung inflammation. The *F508del* mice were divided into four groups and received either vehicles or treatments before LPS challenge. The mice were killed at 24 h to collect plasma. **A**. Plasma lipoxin A4 levels under blockade of PSGL-1. **B**. Plasma lipoxin A4 levels under blockade of P-selectin. **C**. Plasma lipoxin A4 levels under antagonism of PAF. **D**. Plasma lipoxin A4 levels under correction of mutated CFTR trafficking. **E**. Effect of anti-P-selectin on BAL neutrophils. Two-way ANOVA was used for statistical analysis. *P<0.05, *P<0.01 for *F508del* mice receiving treatment versus *F508del* mice receiving vehicle. Four groups of mice showed in each figure were intratracheally challenged with LPS. Values are presented as mean ± SD.

**Figure 6:**
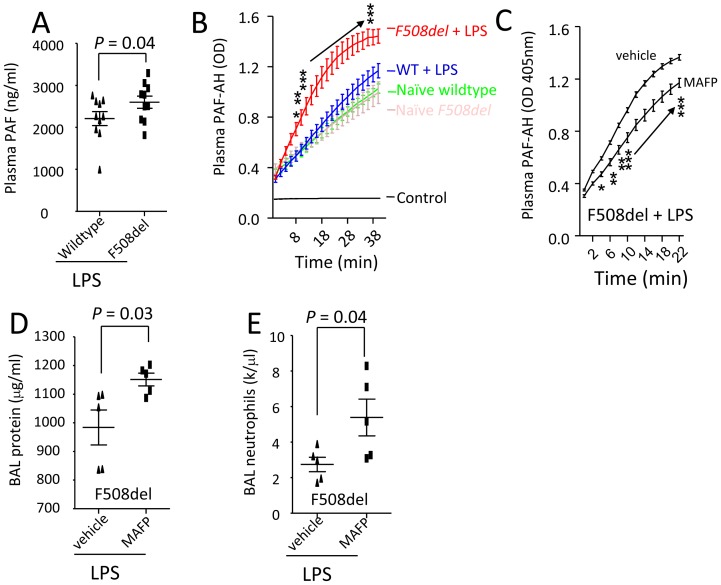
Deficiency of CFTR affects plasma PAF levels and PAF-AH activity in LPS-induced lung inflammation (A–B). Wildtype and *F508del* mice were intratracheally challenged with LPS (5 mg/kg) and killed at 24 h to collect plasma. **A**. Plasma PAF levels were measured by HPLC-MS. **B**. Plasma PAF-AH activity (In control group, PBS was added into reaction system; naïve wildtype and F508del groups, plasma was collected from PBS-treated mice. Repeated measures two-way ANOVA was used for statistical analysis. *P<0.05, **P<0.01, and ***P<0.001 for *F508del* + LPS group versus wildtype + LPS group. **Inhibition of PAF-AH by MAFP worsens acute lung inflammation in** ***F508del*** **mice (C–E)**. *F508del* mice were divided into two groups to receive either vehicle or MAFP (1 mg/kg) treatment 20 min before an intratracheal challenge of LPS (5 mg/kg). The mice were killed at 24 h to collect blood and BAL. **C**. PAF-AH activity. Repeated measures two-way ANOVA was used for statistical analysis. *P<0.05, **P<0.01, and ***P<0.001 for *F508del* mice receiving MAFP versus *F508del* mice receiving vehicle. Both groups were intratracheally challenged with LPS. **D**. BAL protein levels. **E**. BAL neutrophils. Values are presented as mean ± SD. Sample size and *P* values are indicated in each panel.

**Figure 7:**
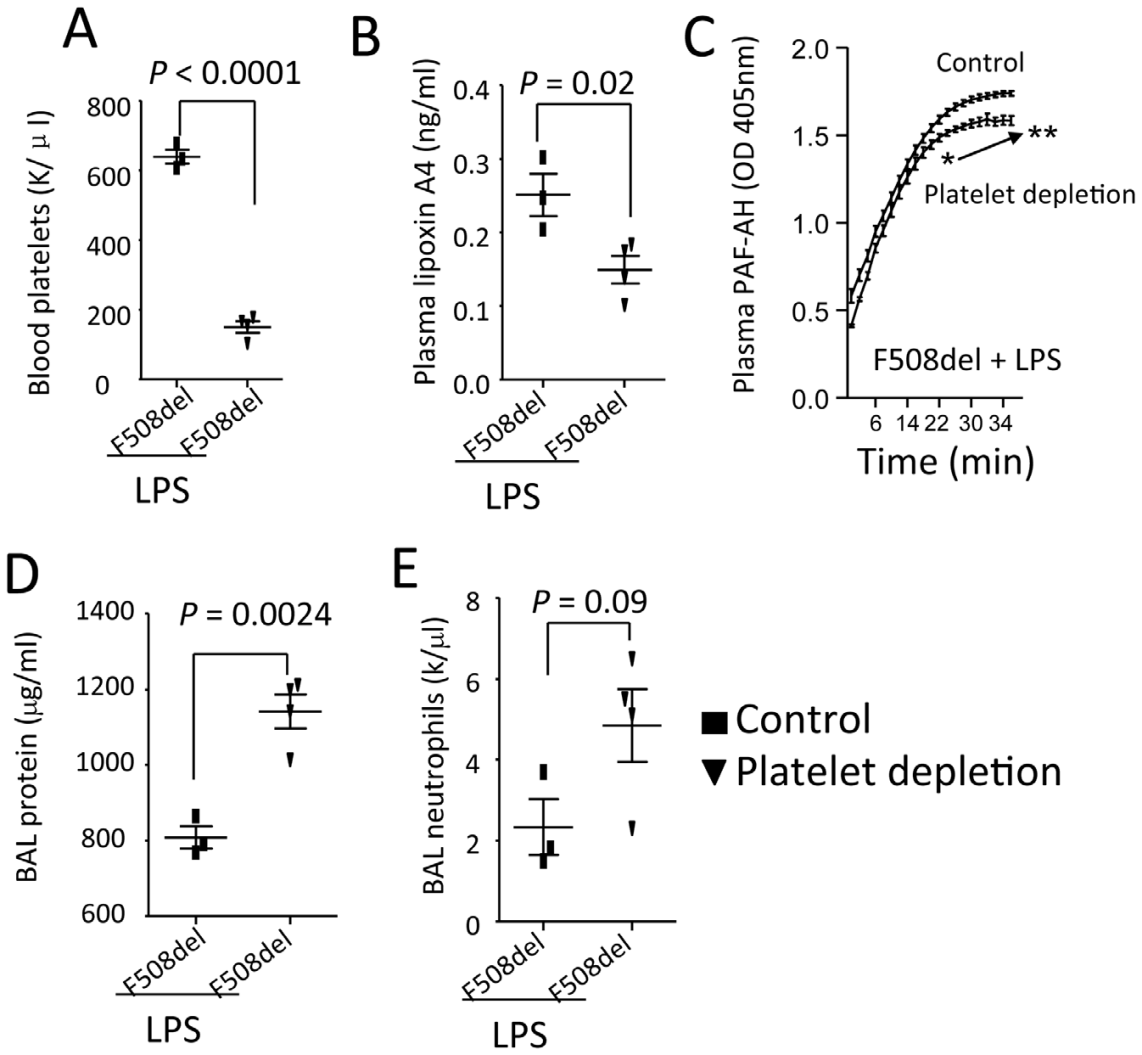
The effects of platelet depletion on plasma lipoxin A4, PAF-AH activity, and lung inflammation in *F508del* mice. *F508del* mice were divided into two groups to receive either control or anti-platelet antibodies. After platelets were depleted, the mice were given an intratracheal challenge of LPS (5 mg/kg) and killed at 24 h to collect blood and BAL. **A**. Change of blood platelet counts. **B**. Plasma lipoxin A4 levels. **C**. Plasma activity of PAF-AH. Repeated measures two-way ANOVA was used for statistical analysis. *P<0.05, **P<0.01 for *F508del* mice receiving anti-platelet antibody versus *F508del* mice control antibody, and both groups were intratracheally challenged with LPS. **D**. BAL protein levels. **E**. BAL neutrophils in LPS-challenged *F508del* mice. Values are presented as mean ± SD. Sample size and *P* values are indicated in each panel.

## Results

### Deficiency of CFTR Reduces Bactericidal Capacity and Promotes Neutrophil Transalveolar Migration and Thrombocytopenia in *E. coli* Pneumonia

To test whether mutation of CFTR would worsen acute lung infection and inflammation, both wildtype and *F508del* mice were intratracheally challenged with *E. coli*. Four hours after *E. coli* challenge, *E. coli* and neutrophil numbers in the BAL collected from pneumonic *F508del* mice were significantly higher compared to wildtype (**Fig. 1** **A and B**), suggesting that CFTR deficiency impairs bactericidal ability in the lung. Blood platelet counts were markedly lower in pneumonic *F508del* mice than wildtype (**Fig. 1C**), suggesting that CFTR deficiency promotes thrombocytopenia during acute lung infection.

### Inhibition of CFTR by MalH-2 Worsens Lung Infection and Inflammation

Considering that mutant CFTR mistrafficking is pathogenic in *F508del* cells, and this process cannot be replicated in the normal cells. Compared to the wildtype, some functions of cells or organs in *F508del* mice may be compromised mice before pathogenic challenge. Administration of CFTR inhibitors is able to quickly induce CFTR malfunction, which could facilitate us to study functional difference in the normal cells or animals. Concurrently, using CFTR inhibitors in the research is able to avoid the genetic differences besides CFTR [10].

To test whether inhibition of CFTR would worsen thrombocytopenia and lung infection, wildtype mice received an ip injection of MalH-2 (a specific CFTR inhibitor) to obtain a CFTR deficiency. Then, MalH-2- or vehicle-treated mice were intratracheally challenged with *E. coli.* Four hours later, blood platelets (**Fig. 2A**) were significantly decreased and plasma MIP-2 levels were higher in MalH-2-treated mice compared to the control (**Fig. 2B**). Also, BAL neutrophils and MIP-2 levels were significantly increased (**Fig. 2C and D**) in CFTR-inhibited mice. These findings suggest that CFTR inhibition promotes thrombocytopenia, MIP-2 production, and neutrophil transalveolar migration during lung infection [15], [20].

### Lipoxin A4 Suppresses MIP-2 Production in CFTR-inhibited LPS-stimulated Neutrophils

The isolated wildtype neutrophils were treated with MalH-2 and then stimulated with LPS to measure MIP-2 levels in the supernatant at 12 h. At this time point, neutrophils were equally viable in both MalH-2 and vehicle treated groups. MIP-2 was markedly increased in MalH-2-treated group, suggesting that inhibition of CFTR in wildtype neutrophils facilitates inflammation (**Fig. 3A**).

To investigate whether p38MAPK or PI3K signaling is involved in CFTR-inhibition mediated MIP-2 production, neutrophils were treated with p38MAPK inhibitor (SB203580) or PI3K inhibitor (Wortmannin) and MalH-2 and then stimulated with LPS to detect MIP-2 at 12 h. The MIP-2 production in CFTR-inhibited LPS-stimulated neutrophils was significantly reduced by SB203580 and boosted by Wortmannin (**Fig. 3A**). These finding suggest that p38MAPK or PI3K signaling could regulate CFTR inhibition-mediated MIP-2 production which reinforces the notion that CFTR has a modulatory effect on inflammatory response in neutrophils.

To examine whether lipoxin A4 would have an anti-inflammatory effect, lipoxin A4 was added to the media of MalH-2-treated LPS-stimulated neutrophils. MIP-2 levels in the supernatant were measured at 12 h. Lipoxin A4 could suppress MIP-2 production dose-dependently compared to vehicle group (**Fig. 3B**), suggesting that lipoxin A4 is a important negative regulator for neutrophil-mediated inflammation.

### Inhibition of CFTR by CFTR_inh_-172 Reduces Ratios of BAL Lipoxin A4/MIP-2 and Lipoxin A4/neutrophil and Worsens Lung Infection

To obtain a CFTR-deficient condition, we treated wildtype mice with CFTR_inh_-172 (a widely-used specific CFTR inhibitor, 3 mg/kg, ip). The CFTR_inh_-172 and vehicle treated mice were intratracheally challenged with *E. coli* and killed at 4 h to perform BAL. BAL lipoxin A4, MIP-2, and neutrophil levels were measured respectively. The ratios of BAL lipoxin A4/MIP-2 (**Fig. 4A**), lipoxin A4/neutrophil (**Fig. 4B**), and MIP-2/neutrophil (**Fig. 4C**) were markedly reduced in the CFTR_inh_-172 treated group compared to vehicle treated group. Higher BAL protein levels (**Fig. 4D**) and *E. coli* numbers (**Fig. 4E**) were present in the CFTR_inh_-172 treated group. Particularly, more *E. coli* colonies were visualized in the BAL proinflammatory cells, especially neutrophils in the CFTR_inh_-172 treated group (**Fig. 4F**).

### Blocking PSGL-1, P-selectin, PAF, or Rectifying Mutated CFTR Trafficking Increases Production of Lipoxin A4 in Circulation in CFTR-deficient Mice

We have reported that blocking PSGL-1, PAF, or rectifying trafficking of mutated CFTR in *F508del* mice could lessen thrombocytopenia and alveolar neutrophil transmigration in the LPS-challenged *F508del* mice [2]. To test whether these interventions would affect plasma lipoxin A4 levels, mice were divided into four groups: wildtype + vehicle, *F508del* + vehicle, wildtype + pretreatment, and *F508del* + pretreatment. After receiving vehicles or interventions, four groups of mice were then given an intratracheal challenge of LPS (5 mg/kg) separately. At 24 h, mice were killed to collect plasma and measure lipoxin A4 levels by ELISA.

We found that blockade of PSGL-1 (by anti-PSGL MAB, 1 mg/kg, iv, **Fig. 5A**), P-selectin (by anti-P-selectin MAB, 1 mg/kg, iv, **Fig. 5B**), or PAF (by its receptor antagonist, WEB2086, 2 mg/kg, iv, **Fig. 5C**), and correction of mutated CFTR trafficking (by corrector KM11060, 2.5 mg/kg, ip) **Fig. 5D**) significantly increased plasma lipoxin A4 levels in the LPS-challenged *F508del* mice compared to the vehicle-treated LPS-challenged *F508del* mice. There was no difference in plasma lipoxin A4 levels among the other groups in each intervention. Concurrently, blockade of P-selectin in the LPS-challenged *F508del* mice could markedly reduce BAL neutrophil counts compared to the control antibody treated *F508del* group (**Fig. 5E**).

### Inhibition of Hydrolysis of PAF by PAF-AH Inhibitor MAFP Worsens LPS-induced Lung Inflammation in CFTR-deficient Mice

PAF-AH is a limiting enzyme for hydrolysis of PAF [21], [22]. Under LPS challenge, both plasma PAF levels and PAF-AH activity were elevated in *F508del* mice compared to the wildtype mice (**Fig. 6A–B**). To test whether PAF-AH is a limiting factor for development of inflammation, two groups of *F508del* mice were ip treated with MAFP (a specific PAF-AH inhibitor [23], 1 mg/kg) 20 min prior to an intratracheal challenge of LPS. Plasma PAF-AH activity was inhibited by MAFP (**Fig. 6C**) at 24 h. BAL neutrophils and protein levels in MAFP-treated *F508del* mice were significantly increased compared to vehicle-treated *F508del* mice (**Fig. 6D–E**), suggesting inhibition of PAF-AH might increase PAF and therefore worsen LPS-induced lung inflammation in the *F508del* mice.

### *F508del* Platelets Play a Role in Regulating Lipoxin A4 Biosynthesis and PAF-AH Activity

To test whether *F508del* platelets play a role in regulating lipoxinA4 production and PAF-AH activity, *F508del* mice were given anti-platelet or control antibodies and then intratracheally challenged with LPS. At 24 h, blood platelets counts (**Fig. 7A**) were decreased in antibody treated group. In platelet-depleted LPS-challenged *F508del* mice, plasma lipoxin A4 (**Fig. 7B**) and PAF-AH activity (**Fig. 7C**) were significantly reduced compared to control antibody treated LPS-challenged wildtype mice, suggesting *F508del* platelets are required for maintaining lipoxin A4 biosynthesis and PAF-AH activity. BAL neutrophils (**Fig. 7D**) and protein levels (**Fig. 6E**) in platelet-depleted LPS-challenged *F508del* mice were increased, suggesting that *F508del* platelets might also play a role in limiting LPS-induced lung inflammation.

## Discussion

Evidence has shown that platelets are activated in the CF patients [7], [24]. Mutation of CFTR causes severe thrombocytopenia in a LPS-induced lung inflammation mouse model. Correction of thrombocytopenia by anti-platelet aggregation, blockade of PSGL-1 or PAF receptor, or rectifying of mutated CFTR trafficking is associated with a better outcome of acute lung inflammation [2]. In this study, for the first time, we found that inhibition of CFTR could mimic CFTR mutation to promote thrombocytopenia during lung infection. These findings support that platelets under CFTR mutation or inhibition are implicated in the pathogenesis of both endotoxin and live bacteria-induced lung inflammation.

The hallmark of airway inflammation in CF patients is a profuse influx of neutrophils into the lungs [4], [25]. Both mutation and inhibition of CFTR could lead neutrophils to producing more proinflammatory cytokines in response to LPS [1]. In CF neutrophils, the recruitment of mutated CFTR to the phagosomes is impaired [26] and this dysfunction affects neutrophil chlorination of phagocytosed bacteria [27]. Here, we found that BAL *E. coli* colonies are markedly increased in CFTR-mutated or inhibited mice compared to wildtype mice. These findings suggest that dysfunction of CFTR reduces bactericidal ability in BAL proinflammatory cells, especially neutrophils and worsens lung infection.

More importantly, in LPS-stimulated neutrophils, pharmacologic inhibition and genetic mutation of CFTR facilitates MIP-2 production and this process is NF-κB-dependent [1]. MIP-2 is a key chemokine that can recruit neutrophils to the airspaces of lung during acute lung inflammation and injury [15], [20]. In this study, we found that inhibition of CFTR could increase MIP-2 production in both blood and BAL and promote neutrophils to transmigrate into the lung. Elevation of MIP-2 production induced by CFTR inhibition in LPS-stimulated neutrophils is regulated positively by p38 MAPK and negatively by PI3K. Lipoxin A4 could dose-dependently inhibit MIP-2 production induced by CFTR inhibition in LPS-stimulated neutrophils. These findings suggest that lipoxin A4 might be a key lipid mediator in limiting MIP-2 production and transalveolar migration of neutrophils.

In fact, BAL lipoxin A4/neutrophil and IL-8/neutrophil ratios in patients with CF were markedly reduced [4]. Lipoxin A4 treatment could suppress of lung neutrophil accumulation and bacterial burden in *P. aeruginosa*-challenged wildtype mice [4]. In line with these findings, we found that inhibition of CFTR could reduce BAL lipoxin A4/MIP-2, lipoxin A4/neutrophil, and MIP-2/neutrophil ratios during lung infection and inflammation. These findings suggest that lack of functional CFTR leads to imbalance of lipoxin A4, which might promote MIP-2 production in the lung, recruit more neutrophils, and deteriorate lung inflammation and infection.

It has to be noted that platelet 12-lipoxygenase is a key enzyme in the biosynthesis of the lipoxins, which are inducible during platelet-neutrophil interaction [5], [6]. Surprisingly, we found that blockade of platelet-neutrophil interaction by PSGL-1 or P-selectin antibodies, or PAF receptor antagonism significantly increased lipoxin A4 production in the circulation and ameliorate the lung inflammation, but the concrete mechanism needs further investigation. Nevertheless, we have revealed that elevation of plasma lipoxin A4 is associated with improvement of lung inflammation in LPS-challenged CFTR-deficient mice.

Furthermore, it has been reported that neutrophils are important generators of PAF when receiving LPS or bacterial challenge [28]. PAF itself or combining with LPS can promote platelet activation and recruitment and worsen endotoxin-induced lung injury [29], [30]. Our study has showed that plasma PAF levels were significantly elevated in LPS-challenged *F508del* mice compared the wildtype mice. PAF receptor antagonism by WEB 2086 could reduce thrombocytopenia and lung inflammation in LPS-challenged *F508del* mice [2]. These findings suggest that PAF might contribute to lung inflammation in CFTR-deficient mice.

Based on the above findings, manipulating PAF might be a promising target for controlling lung inflammation in CFTR-deficient mice. Considering that PAF-AH is a limiting enzyme for hydrolyzing PAF and plasma PAF-AH could limit the development of sepsis [13], we designed experiments to test whether inhibition of PAF-AH affect LPS-induced lung inflammation in the CFTR-deficient mice. Interestingly, we found that plasma PAF-AH activity in LPS-challenged *F508del* mice was increased compared to wildtype mice. MAFP, a specific PAF-AH inhibitor, could significantly reduce PAF-AH activity and therefore worsen lung inflammation in LPS-challenged *F508del* mice. These findings strongly support that PAF is implicated in the development of LPS-induced lung inflammation in CFTR-deficient mice.

In terms that platelets have capacity of secreting the plasma PAF-AH [12] and are implicated in the biosynthesis of lipoxin A4 [5], [6], we used anti-platelet antibody to deplete platelets from peripheral blood and observe the effects of platelet depletion on plasma lipoxin A4, PAF-AH activity, and the outcome of lung inflammation. In these platelets-depleted LPS-challenged *F508del* mice, plasma PAF-AH activity and lipoxin A4 were markedly decreased and concurrently BAL protein levels and neutrophils were increased. These findings support that *F508del* platelets control PAF-AH activity and lipoxin A4 production and contribute to the formation of lung inflammation.

In summary, platelets and neutrophils are key players during LPS or bacteria-induced lung inflammation in CFTR-deficient mice. Lipoxin A4 and PAF are implicated in *E. coli* or LPS-induced lung inflammation in CFTR-deficient mice. Dysfunction of CFTR in the platelets and neutrophils might contribute to abnormalities of lipoxin A4 and PAF in the circulation or airspace of lung during infection and inflammation. Therefore, targeting lipoxin A4 and PAF might be promising avenues to lessening CFTR-deficiency worsened lung inflammation.
